# Beyond Teleconsultation: Exploring the Role of Mobile Health Technologies in Duchenne Muscular Dystrophy

**DOI:** 10.2196/92290

**Published:** 2026-07-21

**Authors:** Eliza Wasilewska, Alessandro Onofri, Andrzej Wasilewski, Jan Wasilewski, Marek Niedoszytko, Jacek Nasiłowski, Sylwia Małgorzewicz

**Affiliations:** 1 Department of Allergology, Medical University of Gdańsk, Marii Skłodowskiej-Curie 3a, Gdańsk, 80-210, Poland; 2 Pediatric Pulmonology & Cystic Fibrosis Unit, Respiratory Intermediate Care Unit, Sleep and Long-Term Ventilation Unit, Bambino Gesù Children's Hospital, Scientific Institute for Research, Rome, Italy; 3 Student Scientific Association of Medical Chemistry and Immunochemistry, Wroclaw Medical University, Marii Skłodowskiej-Curie 48/50, Wrocław, 59-369, Poland; 4 Rochester Institute of Technology, Rochester, NY, United States; 5 Department of Internal Medicine, Pulmonary Diseases and Allergy, Medical University of Warsaw, Warsaw, Poland; 6 Department of Lung Diseases, Mazovian Centre for Lung Diseases and Tuberculosis, Otwock, Poland; 7 Department of Clinical Nutrition, Medical University of Gdańsk, Debinki, Gdańsk, 80-211, Poland, 48 6016776

**Keywords:** Duchenne muscular dystrophy, DMD, remote monitoring, mobile health technologies, mobile health, mHealth

## Abstract

Duchenne muscular dystrophy (DMD) is a progressive, multisystem disease requiring long-term monitoring of respiratory, cardiac, and functional status. Advances in care have extended survival, increasing the need for continuous, coordinated management. In parallel, digital health technologies have enabled remote monitoring and data collection outside traditional clinical settings. This Viewpoint examines the role of device-based remote monitoring in DMD and argues that it should be understood as an emerging multisystem digital surveillance framework rather than a collection of isolated technologies. This Viewpoint is based on a synthesis of published literature on device-based remote monitoring and digital health technologies in DMD and experiences at our center. The aim was to outline the current clinical and technological landscape and support an interpretive perspective. Device-based monitoring in DMD encompasses a range of technologies, including home spirometry, ventilator-integrated telemonitoring, wearable activity sensors, cardiac rhythm monitoring, and interactive rehabilitation systems. These approaches enable longitudinal assessment of physiological and functional parameters in real-world settings. However, the current evidence base is heterogeneous and largely limited to feasibility studies, small cohorts, and extrapolated data. While data acquisition is technically feasible and increasingly available, integration into clinical workflows remains constrained by the lack of validated digital biomarkers, standardized monitoring frameworks, and interoperable data systems. Remote monitoring in DMD is evolving toward a connected, multisystem model of disease surveillance. Its clinical impact will depend on the validation of digital end points, development of decision support frameworks, and integration into multidisciplinary care pathways. Bridging the gap between data generation and clinical decision-making remains a key priority for future research and implementation. These findings support a shift from isolated technologies toward a coordinated, clinically integrated model of multisystem care in DMD.

## Introduction

Duchenne muscular dystrophy (DMD) is a progressive neuromuscular disorder with multisystem involvement, including loss of motor function, respiratory insufficiency, and cardiomyopathy [1-3]. Advances in multidisciplinary care have prolonged survival and altered the clinical profile of the disease, resulting in a growing population of adolescents and adults living with long-term respiratory, cardiac, and functional complications [2,4]. This shift increases the need for long-term monitoring, technology-assisted support, and coordinated chronic care.

Traditionally, the management of patients with DMD has relied on periodic visits to specialized centers [1,2]. The COVID-19 pandemic highlighted the vulnerability of this model by limiting access to essential assessments and accelerating the implementation of telemedicine solutions that helped maintain continuity of care [5].

Mobile health solutions and wearable sensor–based systems are already being developed and implemented in other areas of chronic disease management, enabling remote acquisition of physiological data, monitoring of vital parameters, and analysis of long-term health trends. Such approaches are already used in areas including cardiology, metabolic disorders, and activity monitoring, demonstrating that continuous data collection outside the clinical setting is becoming an important component of modern health care systems [6-12].

This approach shifts attention from episodic assessment toward longitudinal observation of disease-related changes in the patient’s everyday environment. In a condition characterized by gradual, multisystem progression, the ability to capture trends over time may complement conventional clinic-based evaluations and broaden the basis for clinical decision-making.

From a digital health perspective, DMD may be regarded as a model condition for the development of connected care in rare, high-risk, and technology-dependent disorders. In our previous work, we described the use of telemedicine in DMD primarily from the perspective of remote consultations, communication pathways, and organizational aspects of care delivery [13].

While previous work has primarily focused on telemedicine as a means of remote communication and service delivery, the device-based dimension of digital health introduces a distinct paradigm shift. Rather than supporting isolated clinical encounters, connected monitoring systems enable continuous, longitudinal observation of disease processes across multiple organ systems.

In this Viewpoint, we propose that device-based remote monitoring in DMD should not be understood as a collection of individual technologies but rather as an emerging multisystem digital surveillance framework. This perspective shifts the focus from the feasibility of individual tools toward their integration into coordinated clinical pathways and decision-making processes.

From this perspective, digital health in DMD extends beyond telemedicine as a communication tool and introduces new models of continuous, data-driven care. The aim of this Viewpoint is to examine the role of device-based remote monitoring in DMD and argue that it represents an emerging multisystem digital surveillance framework rather than a collection of isolated technologies. This Viewpoint advances three key messages: (1) remote monitoring in DMD should be viewed as a multisystem digital framework rather than as isolated tools; (2) clinical integration represents an important challenge, although not necessarily the single dominant barrier; and (3) implementation requires interoperable, multidisciplinary care models.

## Approach and Evidence Overview

This Viewpoint is based on a synthesis of published literature on device-based remote monitoring and digital health technologies in DMD and experiences at our center. The aim was to outline the current clinical and technological landscape and support an interpretive perspective.

Relevant literature was identified through targeted searches of PubMed, Scopus, and Google Scholar conducted between November 2 and 30, 2025. Search terms included combinations of keywords related to DMD, telemonitoring, wearable sensors, home spirometry, ventilatory support, cardiac monitoring, activity monitoring, and digital or virtual rehabilitation. The search process was iterative and complemented by manual screening of reference lists from relevant publications.

Consistent with the conceptual scope of this Viewpoint, no formal inclusion or exclusion criteria, structured screening process, or risk-of-bias assessment were applied. Studies were selected based on their relevance to device-based monitoring, representation of current technological approaches, and potential clinical applicability in DMD.

The evidence base included feasibility studies, pilot investigations, observational reports, and selected implementation studies. In addition to DMD-specific data, evidence from broader populations with neuromuscular disorders was considered when it was judged to be clinically transferable. Clinical transferability was defined as applicability to shared pathophysiological domains, such as respiratory insufficiency, ventilatory support, cardiac involvement, or functional decline, as well as the use of comparable monitoring approaches and relevance to clinical practice in DMD. A substantial proportion of the available evidence is derived from broader neuromuscular or other chronic disease populations. While such findings may be clinically informative, their applicability to DMD remains indirect and should be interpreted with caution.

The identified evidence was synthesized narratively and organized into respiratory, cardiac, and functional and rehabilitation domains. Given the heterogeneity of study designs, devices, and reported outcomes, a structured systematic comparison or quantitative synthesis was not undertaken. Multimedia Appendix 1, [14-47] provides a detailed overview of the included studies, including study design, population, and evidence source.

## Types of Remote and Mobile Health Technologies in DMD

### Overview

Remote monitoring in DMD comprises a diverse set of technologies differing in clinical purpose, data type, and integration with therapeutic devices. Rather than a single solution, digital health in DMD forms a layered ecosystem that extends assessment beyond clinic visits and enables collection of longitudinal real-world data. For clarity, these technologies can be grouped into several functional categories consistent with broader frameworks in wearable and mobile health for chronic diseases [5,12]. Table 1 summarizes the current evidence base across domains, highlighting the heterogeneity of available data and the limited proportion of DMD-specific studies.

**Table 1.** Summary of evidence on device-based remote monitoring in Duchenne muscular dystrophy (DMD)^a^.

Domain and technology	Study type	Population	Evidence source	Key findings	Limitations
Respiratory					
Home spirometry	Pilot studies	Patients with DMD	DMD-specific evidence	Suggests feasibility for home monitoring and enables longitudinal tracking of lung function	Small sample sizes, short follow-up, and limited outcome data
Ventilator-integrated telemonitoring	Observational studies	Patients with neuromuscular disorders	Extrapolated from the neuromuscular population	Indicates potential for remote adjustment of ventilation and adherence monitoring	Limited DMD-specific evidence and heterogeneous data
Telemonitoring in respiratory care	RCTs^b^ and observational studies	COPD^c^ and mixed populations	Extrapolated from other chronic conditions	Suggests potential reduction in hospitalizations and improvement in care coordination	Not disease specific and limited applicability to DMD
Cardiac					
Wearable ECG^d^ and rhythm monitoring	Feasibility and observational studies	Mixed populations	Extrapolated from cardiology and mixed cohorts	Demonstrates feasibility of remote rhythm monitoring and arrhythmia detection	Limited validation in DMD and unclear impact on outcomes
Implantable cardiac devices (CIEDs^e^)	Case series and retrospective studies	Patients with DMD	DMD-specific evidence	Provides continuous rhythm monitoring alongside therapeutic support	Limited evidence base, small cohorts, and invasive nature
Functional					
Activity trackers and accelerometers	Observational studies	Patients with DMD	DMD-specific evidence	Suggests association with functional status and disease progression	Lack of standardized end points and variability in metrics
Wearable motion sensors	Pilot studies	Patients with DMD	DMD-specific evidence	Enables real-world monitoring of mobility and daily activity	Small samples and limited longitudinal validation
Rehabilitation					
Virtual reality and digital rehabilitation	Pilot studies and RCTs	Patients with DMD	DMD-specific evidence	Suggests improved engagement and motor task performance	Heterogeneous designs and small sample sizes
Cross-domain					
Integrated digital monitoring systems	Feasibility studies	Mixed populations	Extrapolated from multiple clinical domains	Enables multisystem data collection and remote care models	Lack of interoperability and unclear clinical pathways

a This table reflects the predominance of small-scale and feasibility-based studies, as well as the frequent reliance on evidence extrapolated from non-DMD populations.

b RCT: randomized controlled trial.

c COPD: chronic obstructive pulmonary disease.

d ECG: electrocardiogram.

e CIED: cardiac implantable electronic device.

### Physiological Monitoring

These systems measure vital signs and organ-specific parameters in the home setting, including respiratory and cardiac function. Data may include lung function indexes, oxygen saturation, heart rate, rhythm-related measures, and other indicators of organ performance. Their main purpose is to identify trends, early deterioration, or deviations from baseline that might be missed during periodic clinical assessments.

### Functional and Activity Monitoring

This category includes tools that assess motor performance, mobility, and daily activity using wearable sensors and motion-tracking methods. Metrics such as step count, activity level, movement patterns, and postural transitions reflect how patients function in everyday environments. In DMD, such data help characterize functional trajectories, fatigue-related fluctuations, and the real-world impact of interventions [11].

### Therapy-Integrated Monitoring

Some monitoring functions are embedded within therapeutic devices, where data collection occurs as part of treatment. These systems record use patterns, performance parameters, and adherence information. They provide insights into treatment effectiveness and evolving patient needs, linking therapy and monitoring in continuous feedback loops that support individualized care.

### Rehabilitation and Interactive Digital Systems

Digital rehabilitation platforms combine exercise or training with performance data capture. Often using interactive interfaces, they support therapeutic engagement while tracking task completion, intensity, and progression. Beyond their rehabilitative role, they contribute structured information on functional status over time [5,11].

### Summary

Together, these categories show that remote monitoring in DMD involves complementary systems addressing physiological status, functional performance, therapy use, and rehabilitation engagement, forming an integrated approach to long-term disease management. Across domains, the available evidence remains heterogeneous and is dominated by small, feasibility-focused studies.

## Clinical Application of Remote Monitoring in DMD

### Overview

The conceptual domains outlined above translate into clinical application across the organ systems affected in DMD. This transition from technological categories to clinical use highlights how remote monitoring functions as an integrated component of multisystem care. Because disease progression in DMD affects respiratory, cardiac, motor, and functional systems in parallel, remote monitoring approaches support surveillance, therapy optimization, and longitudinal assessment across these interconnected areas.

### Respiratory System

#### Overview

Progressive respiratory muscle weakness in DMD leads to a gradual reduction in lung volumes, ineffective cough, and increasing risk of respiratory complications. With disease progression, patients ultimately develop chronic respiratory insufficiency requiring ventilatory support. Because both pulmonary function and ventilatory therapy parameters change over time and may not be fully captured during periodic clinic visits, respiratory care has become an important focus of remote monitoring approaches in DMD.

#### Pulmonary Function Tests

Pulmonary function testing represents the primary clinical method for quantifying respiratory decline in DMD. Standards of care recommend testing from approximately 5 to 6 years of age, with reassessment every 6 to 12 months depending on disease stage [2]. However, clinic-based spirometry provides only episodic snapshots of respiratory function and may fail to capture short-term fluctuations in respiratory status. This gap has driven the development of home respiratory monitoring technologies.

One of the earliest examples was the use of portable electronic peak flow meters during the Duchenne Muscular Dystrophy Long-term Idebenone Study (DELOS) trial, in which boys with DMD performed daily peak expiratory flow recordings at home, with the data downloaded during scheduled clinic visits [15]. Although not connected in real time, the study demonstrated both feasibility and patient adherence to home pulmonary monitoring.

More advanced home spirometry systems were evaluated in the e-PULMoDMD (e-Monitoring of Pulmonary Function in Patients With Duchenne Muscular Dystrophy at Home) study using the AioCare spirometer linked to a smartphone app [16,17]. These platforms enabled home measurement of forced vital capacity and forced expiratory volume in 1 second, provided step-by-step instructions, and delivered immediate feedback through graphical displays of spirometry curves. Some patients perceived daily recordings as a form of respiratory exercise, suggesting an additional motivational component [17]. Available evidence for home spirometry in DMD is primarily derived from small feasibility and pilot studies typically involving limited patient cohorts and short follow-up periods. While these studies consistently demonstrate technical feasibility and patient adherence, they provide limited data on clinical outcomes or long-term impact on disease management.

#### Ventilation Support

With disease progression, many patients with DMD require noninvasive ventilation (NIV) and, less frequently, invasive mechanical ventilation. Both modalities require ongoing monitoring to ensure adequate ventilation, comfort, and adherence. Early ventilator systems allowed for only manual data extraction [18], whereas modern platforms offer internal or external modems capable of transmitting use time, leak values, respiratory rate, tidal volume, inspiratory-to-expiratory ratios, and longitudinal trends of key parameters [19].

Telemonitoring has been associated with clinical benefits in broader neuromuscular populations; however, evidence specific to DMD remains limited, and the extent to which these findings translate to DMD care is uncertain. Bertini et al [18] demonstrated that weekly remote review combined with 24-hour support reduced emergency visits and hospital admissions among ventilated children. Trucco et al [19] reported high caregiver satisfaction and fewer hospital presentations in children using NIV, including those with DMD. During the COVID-19 pandemic, Onofri et al [20] showed that remote interpretation and adjustment of ventilatory parameters were feasible, and none of the telemonitored children required urgent admission. Additional reports from broader neuromuscular populations describe improved clinical stability, reduced hospitalization rates, and enhanced caregiver confidence associated with respiratory telemonitoring [7,10]. Despite these benefits, routine implementation remains limited by infrastructural, regulatory, and reimbursement barriers. Evidence supporting ventilator-based telemonitoring is stronger but largely extrapolated from broader neuromuscular populations rather than DMD-specific cohorts. Reported benefits include reduced hospitalizations and improved caregiver confidence; however, data specific to DMD remain limited and heterogeneous.

#### Summary of the Respiratory Domain

Respiratory remote monitoring in DMD includes home spirometry and telemonitoring integrated with ventilatory support. These approaches allow for the collection of longitudinal data on pulmonary function and therapy use outside routine clinic visits, although current evidence is based mainly on small studies and feasibility reports.

### Cardiac System

#### Overview

Cardiac involvement in DMD most commonly manifests as dilated cardiomyopathy and rhythm disturbances and represents a major source of morbidity and mortality [2]. Cardiac status may deteriorate gradually and remain clinically silent, which makes regular surveillance using echocardiography, electrocardiography, cardiac biomarkers, and cardiac magnetic resonance imaging a key component of care [3]. Because conventional follow-up relies on periodic, center-based investigations, dynamic changes in ventricular function or intermittent arrhythmias may not be fully captured between visits, supporting interest in remote and device-based cardiac monitoring approaches.

In more advanced stages, management extends beyond surveillance and includes cardioprotective pharmacotherapy as well as, in selected cases, implantable cardiac devices. Therefore, monitoring may occur both through dedicated wearable systems and as an integrated function of therapeutic devices, linking remote observation with active treatment.

#### Cardiac Rhythm Monitoring

Noninvasive cardiac telemonitoring is most commonly delivered through wearable sensors and ambulatory rhythm monitoring systems, often incorporated within broader telehealth models. Wearable heart rate devices, chest straps, patch-based electrocardiographic monitors, and consumer-grade smartwatches have demonstrated feasibility for rhythm screening and symptom correlation in pediatric and chronic disease populations [8]. These technologies enable intermittent or continuous recording of heart rate and rhythm outside the clinical setting and illustrate the technical feasibility of long-term rhythm surveillance.

Experience with ambulatory rhythm monitoring in DMD also includes conventional Holter-based assessments reported in small observational studies [48]. Nevertheless, the use of wearable or ambulatory rhythm monitoring specifically in DMD remains limited. Available studies are few, involve small sample sizes, and lack definitive evidence linking remote rhythm surveillance with changes in management or clinical outcomes. As a result, wearable cardiac monitoring in DMD currently represents a developing component of digital health rather than an established standard. Current evidence for wearable cardiac monitoring in DMD remains limited and is derived mainly from small observational studies and feasibility reports.

#### Implantable Cardiac Devices

In more advanced stages of cardiomyopathy, implantable cardioverter defibrillators (ICDs) and, less frequently, pacemakers or cardiac resynchronization therapy systems may be considered. Although primarily therapeutic, these devices also generate continuous monitoring data, blurring the boundary between treatment and remote surveillance. Published experience with pacemakers, cardiac resynchronization therapy, and ICDs in DMD consists mainly of small case series and retrospective reports, reflecting cautious use and uncertainty regarding long-term benefit in this population [21-24]. Additional reports describe ICD implantation in selected patients, with automatic rhythm surveillance and event logging as inherent device functions, although the structured use of remotely transmitted data in DMD care has not been systematically evaluated [25,26].

Left ventricular assist device implantation in DMD has been described only in isolated case reports and small series, involving devices such as HeartMate II, HeartWare, and Jarvik systems [25-28]. These cases highlight complex ethical and practical considerations in patients with advanced neuromuscular disability and respiratory compromise. Although left ventricular assist device controllers technically allow for remote review of device alarms and selected performance parameters, there are no data confirming their integrated use within structured remote cardiac follow-up in DMD. Evidence regarding implantable cardiac devices in DMD is based primarily on case series and retrospective analyses. While these devices provide continuous monitoring capabilities, their role in structured remote monitoring and long-term outcome improvement in DMD remains insufficiently defined.

#### Cardiac Parameters Within Telehealth Models

Cardiac parameters have also been monitored indirectly within multisystem telemedicine programs. Trucco et al [19] reported that telehealth follow-up originally implemented for NIV in children, including those with DMD, facilitated remote triage of both respiratory and cardiac symptoms and was associated with fewer emergency consultations. These findings suggest that telecardiology in DMD may be particularly effective when embedded within integrated, multidisciplinary monitoring models rather than functioning as a stand-alone tool.

#### Summary of the Cardiac Domain

Cardiac remote monitoring in DMD currently involves wearable or ambulatory rhythm surveillance, data generated by implantable therapeutic devices, and cardiac parameters captured within broader telehealth programs. Although a range of technologies is available, their structured clinical use in DMD remains limited. The evidence base consists primarily of small case series, feasibility studies, and data extrapolated from broader cardiology populations, which limits the strength of conclusions regarding clinical impact in DMD.

### Rehabilitation and Functional Monitoring

#### Overview

Progressive loss of motor function is a defining feature of DMD and represents a key target for digital and device-based monitoring approaches. Functional decline evolves gradually and may fluctuate with fatigue, intercurrent illness, or changes in therapy. Traditional clinical assessments—such as timed tests, walking tests, or structured upper-limb scales—provide standardized but episodic measurements obtained under supervised conditions and may not fully reflect how patients function in everyday environments. These characteristics make functional performance a natural target for digital technologies that enable repeated observation, objective quantification, and technology-supported rehabilitation.

#### Activity Monitoring

Wearable sensor technologies have enabled continuous or repeated measurement of real-world physical activity in individuals with DMD. Accelerometers, actigraphy devices, and magneto-inertial sensor systems have been used to quantify daily movement patterns, walking behavior, and upper-limb use outside the clinic [29-32]. These approaches shift assessment from performance in a controlled test toward observation of habitual activity in natural settings.

Subsequent studies have extended these methods to community walking and upper-extremity activity, demonstrating feasibility and correlations between sensor-derived metrics and established functional measures [33,34]. Broader activity profiling—including in nonambulant individuals—has shown that digital activity metrics can capture dimensions of participation and movement not represented by traditional ambulation-focused scales [35,36]. Associations between physical activity patterns and physiological parameters further suggest that movement data may reflect overall clinical status [37].

Despite this promise, barriers remain to routine clinical adoption. Long-term adherence, variability in device placement, and the need for clinically interpretable metrics pose challenges. From a regulatory perspective, the qualification of wearable-derived end points in DMD is still evolving, with ongoing discussion regarding validity, reliability, and clinical meaning of digital mobility outcomes. Although wearable technologies enable detailed characterization of real-world activity, the evidence remains largely exploratory. Studies are typically small and heterogeneous, and the relationship between digital activity metrics and clinically meaningful outcomes in DMD is not yet fully established.

#### Respiratory Rehabilitation

While digital rehabilitation in DMD has largely focused on motor function, technology-assisted rehabilitation of the respiratory system has been explored far less frequently. An early example is the computerized respiratory muscle training described by Vilozni et al [38] in which visual computer feedback was used to guide breathing exercises in children with DMD. Although this approach was not based on immersive virtual environments and was not designed for remote use, it illustrates that digital tools have also been applied to support functional training of respiratory muscles. However, compared with the expanding field of interactive motor rehabilitation platforms, digital respiratory rehabilitation remains markedly underdeveloped.

#### Virtual Reality and Interactive Motor Rehabilitation

Beyond passive monitoring, digital technologies are also used to actively support rehabilitation. Earlier exploratory platforms further illustrate the feasibility of immersive or augmented environments in neuromuscular populations [39,40]. Virtual reality (VR), game-based platforms, and interactive computer or smartphone systems combine therapeutic exercises with real-time feedback and data capture. In DMD, these approaches have been explored mainly for upper-limb training and engagement in individuals with limited mobility [41-43].

Controlled and crossover studies have reported that VR-based or interactive training can improve task performance, support motor learning, enhance motivation, and increase adherence to therapeutic activities [44,45,49]. These systems often record performance parameters such as movement accuracy, task completion, and interaction patterns, generating structured data alongside therapy. A systematic review highlights the growing but still heterogeneous evidence base for VR technologies in DMD [50].

However, most VR and interactive systems are not yet calibrated against validated clinical end points, and study sizes remain small. A key future direction is linking digitally captured performance data with established functional scales and clinically meaningful thresholds, as well as integrating remote supervision and data sharing with rehabilitation teams. Evidence supporting virtual and interactive rehabilitation in DMD is growing but remains limited by small sample sizes, variability in study design, and lack of standardized outcome measures. As a result, translation of these approaches into routine clinical practice is still constrained.

#### Summary of the Rehabilitation and Functional Domain

Digital approaches to rehabilitation in DMD encompass 2 complementary directions: wearable systems that capture real-world movement and activity and interactive platforms that combine therapy with structured data generation. Together, they extend functional assessment beyond clinic-based testing and provide insights into everyday performance and engagement. In contrast, technology-assisted respiratory rehabilitation has received limited attention, highlighting an imbalance in the development of digital therapeutic strategies across organ systems.

## Discussion

### Data Generation

Remote and device-based monitoring in DMD is often presented as a collection of emerging technologies; however, this perspective underestimates its structural implications for clinical care. Rather than functioning as isolated tools, these systems collectively represent an evolving digital surveillance architecture that extends disease monitoring beyond episodic clinical encounters. Recent health system disruptions have accelerated adoption of remote infrastructures, but technological availability has progressed faster than the development of unified clinical frameworks [7].

A central challenge is the asymmetry between data generation and clinical embedding. Connected devices can reliably capture physiological, behavioral, and therapy-related parameters, yet structured pathways translating these signals into clinical decisions remain insufficiently defined. Therefore, the limitation is not measurement capability but the absence of harmonized interpretation models, escalation algorithms, and clinically validated decision thresholds linked to digital outputs [6,12]. Without such frameworks, digital metrics risk remaining descriptive rather than decision supportive. For clinicians, this means that most device-generated metrics currently inform clinical awareness rather than directly triggering therapeutic decisions.

Meaningful transformation depends on interoperable data ecosystems. Device development alone does not ensure system-level impact; integrated data flows, standardized formats, and analytical layers capable of aggregating multisystem inputs are required. Because DMD care is inherently multidisciplinary, digital monitoring must support coordinated oversight rather than parallel, organ-specific data silos. Therefore, interoperability becomes a clinical requirement, not only a technical feature. This highlights a fundamental gap between technological capability and clinical implementation. Without clearly defined response pathways, digital monitoring risks remaining an observational layer rather than an active component of care. Bridging this gap requires not only validation of digital metrics but also their integration into routine clinical workflows and decision-making processes. This limitation is further compounded by the fact that much of the available evidence is derived from small-scale or non–DMD-specific studies, which restricts the ability to define robust clinical thresholds and validate digital end points.

### Convergence of Monitoring and Therapy

An additional structural shift concerns the integration of monitoring with therapy. Ventilatory devices and implantable cardiac systems already combine treatment delivery with continuous data logging, illustrating convergence between therapeutic and surveillance functions [19]. A similar convergence is emerging in interactive rehabilitation platforms that generate performance metrics during therapy [11]. While this model supports individualized care, it also introduces challenges related to data volume, workflow integration, and clinician response capacity. Sustainable implementation will require automated preprocessing, clinically meaningful dashboards, and predefined response protocols.

Development remains uneven across domains. Some areas demonstrate technologically mature solutions, whereas others still lack validated digital end points or structured follow-up models. This imbalance reflects differences in research maturity rather than differences in clinical relevance, highlighting the need for coordinated development and validation strategies across organ systems [50-57].

### Regulatory and Reimbursement Dependencies

The scalability of digital monitoring is dependent on regulatory recognition and reimbursement alignment. Without formal acceptance of digital biomarkers and monitoring outputs within clinical and funding frameworks, remote systems remain adjunctive rather than foundational. Standardization of data governance, privacy models, and medicolegal responsibility is similarly necessary for widespread implementation [58].

Taken together, these observations suggest that clinical integration represents a key challenge in digital health for DMD alongside other interrelated factors such as limited evidence, lack of validated end points, and system-level constraints. Without embedding device-generated data into structured clinical workflows, digital monitoring risks remaining descriptive rather than actionable. Therefore, future progress will depend less on the development of new technologies and more on the effective integration of existing systems within interoperable, multidisciplinary care models.

Digital monitoring in DMD is transitioning from feasibility-driven projects to a connected model of multisystem surveillance and therapy support. Future progress is likely to depend on the validation of digital end points, development of interoperable systems, and integration of decision support frameworks within multidisciplinary care pathways.

### Limitations

This Viewpoint adopted a narrative and conceptual approach rather than a systematic review methodology. The underlying evidence base across digital monitoring technologies in DMD is heterogeneous, consisting largely of feasibility studies, pilot investigations, and small observational cohorts using diverse devices, metrics, and follow-up periods. As a result, neither direct comparison between technologies nor quantitative synthesis of outcomes was feasible. In addition, rapid technological evolution means that device capabilities and integration models may outpace the published literature. These limitations reflect the developmental stage of digital monitoring in DMD rather than weaknesses of individual systems.

### Future Directions

Future development of digital health technologies in DMD will depend not only on technological progress but also on clinical validation, system-level integration, and adaptation of regulatory and reimbursement frameworks. A key priority is device interoperability and the development of integrated platforms enabling multidisciplinary clinical oversight. In parallel, the implementation of analytical tools that support clinical decision-making, along with prospective studies evaluating the impact of remote monitoring on clinical outcomes, quality of life, and resource use, will be essential. Effective implementation of these solutions will also require sustainable funding mechanisms that recognize remote monitoring as an integral component of standard care for patients with DMD.

### Conclusions

Remote monitoring in DMD is evolving from the use of individual devices toward a more coordinated, multisystem model of digital disease surveillance. While current applications demonstrate technical feasibility across respiratory, cardiac, and functional domains, their integration into clinical practice remains limited.

An important challenge lies not in the ability to generate data but in the absence of validated digital biomarkers, standardized monitoring frameworks, and clearly defined clinical response pathways. As a result, most device-generated data currently support clinical awareness rather than directly informing decision-making.

Future progress is likely to depend on the validation of clinically meaningful digital end points, the development of interoperable data systems, and the integration of remote monitoring into multidisciplinary care pathways. Addressing these challenges will be important for enabling a transition of digital health in DMD from an adjunctive approach toward a more integrated component of disease management. However, given the current limitations of the evidence base, the clinical impact of these approaches remains to be established.

## Supplementary material

10.2196/92290 Multimedia Appendix 1 Clinical and feasibility studies on device-based remote monitoring, wearable technologies, and digital rehabilitation in Duchenne muscular dystrophy and related populations.

## Abbreviations

DELOS: Duchenne Muscular Dystrophy Long-term Idebenone Study
DMD: Duchenne muscular dystrophy
e-PULMoDMD: e-Monitoring of Pulmonary Function in Patients With Duchenne Muscular Dystrophy at Home
ICD: implantable cardioverter defibrillator
NIV: noninvasive ventilation
VR: virtual reality
