# Supplementary material for: Beyond Teleconsultation: Exploring the Role of Mobile Health Technologies in Duchenne Muscular Dystrophy
Source: JMIR Form Res. 2026 Jul 21;10:e92290. doi: 10.2196/92290 (PMC13387638; doi:10.2196/92290)
Supplement: Multimedia Appendix 1 [file formative-v10-e92290-s001.docx]

**Supplementary Table 1. Clinical and feasibility studies on device-based remote monitoring, wearable technologies, and digital rehabilitation in Duchenne muscular dystrophy (DMD) and related populations.**

| Ref | **Authors** | **Year** | \| **Technology / Application** \| \| --- \| |
| --- | --- | --- | --- | --- |
| **PULMONOLOGY** | | | |
| [15] | Buyse GM et al. | 2018 | Home-based pulmonary function monitoring |
| [16] | Wasilewska E et al. | 2021 | Home spirometry |
| [17] | Wasilewska E et al. | 2022 | Telemonitoring of pulmonary function |
| [18] | Bertini S et al. | 2012 | Telemonitoring of home ventilation |
| [19] | Trucco F et al. | 2019 | Telemonitoring of non-invasive ventilation |
| [20] | Onofri A et al. | 2021 | Telemedicine in home ventilation |
| **CARDIOLOGY** | | | |
| [21] | Groh WJ et.al. | 2015 | Ambulatory Holter monitoring |
| [47] | Fayssoil A et al. | 2010 | Pacemaker implantation |
| [14] | Palladino A et al. | 2019 | ICD / CRT-D devices |
| [22] | Kono T et al. | 2015 | CRT-D implantation |
| [23] | Fayssoil A et al. | 2016 | Implantable devices (PM, CRT, CRT-D) |
| [24] | Villa C et al. | 2022 | ICD in paediatric cardiology |
| [25] | Ryan TD et al. | 2014 | Implantable LVAD (HeartMate II device and Heart Ware device) |
| [26] | Iodice F et al. | 2015 | Implantable LVAD (Jarvik 2000) |
| [27] | Perri G et al. | 2017 | Implantable LVAD (destination therapy) |
| [28] | Stoller D et al. | 2017 | Implantable LVAD |
| **FUNCTIONAL / WEARABLE MONITORING** | | | |
| [29] | Jeannet PY et al. | 2011 | Wearable accelerometer and a gyroscope. |
| [30] | Kimura S et al. | 2014 | Wearable wrist actigraph (Motion logger Watch) |
| [31] | Davidson ZE et al. | 2015 | Wearable Accelerometer. |
| [32] | Le Moing AG et al. | 2016 | Wearable devices (accelerometer, gyroscope, magnetometer) |
| [33] | Fowler EG et al. | 2018 | Wearable Accelerometer. |
| [34] | van der Geest A et al. | 2020 | Wearable Accelerometer. |
| [35] | Killian M et al. | 2020 | Wearable Accelerometer. |
| [36] | Arteaga D et al. | 2020 | Wearable Accelerometer. |
| [37] | Kaslow JA et al. | 2023 | Wearable Accelerometer. |
| **VIRTUAL REALITY** | | | |
| [38] | Vilozni D et al. | 1994 | Video games. |
| [46] | Correa AGD et al. | 2009 | VR games |
| [39] | Hashimoto Y et al. | 2010 | EEG-based brain-computer interfaces (BCIs) and VR for communication |
| [40] | Malheiros SRP et al. | 2016 | Computer games. |
| [41] | Capelini CM et al. | 2017 | Smartphone-based rehabilitation |
| [42] | Heutinck L et al. | 2018 | VR computer game. |
| [43] | Massetti T et al. | 2018 | VR computer games. |
| [44] | de Freitas BL et al. | 2019 | VR computer games. |
| [45] | Quadrado VH et al. | 2019 | VR Computer games. |

References:

14. Palladino A, Papa AA, Morra S et al. Are there real benefits to implanting cardiac devices in patients with end-stage dilated dystrophinopathic cardiomyopathy? Review of literature and personal results. Acta Myol. 2019 Mar 1;38(1):1-7. PMID: 31309174; PMCID: PMC6598406.

15. Buyse GM, Rummey C, Meier T, et al. Home-based monitoring of pulmonary function in patients with Duchenne muscular dystroph. J Neuromuscul Dis. 2018;5(4):419-430. [doi: 10.3233/JND-180338] [Medline: 30282375]

16. Wasilewska E, Sobierajska-Rek A, Małgorzewicz S, et al. Home e-monitoring of pulmonary function in Duchenne muscular dystrophy during the COVID-19 pandemic: a pilot study. Int J Environ Res Public Health. 2021;18(17). [doi: 10.3390/ijerph18178967]

17. Wasilewska E, Sobierajska-Rek A, Małgorzewicz S et al. Benefits of telemonitoring of pulmonary function-3-month follow-up of home electronic spirometry in patients with Duchenne muscular dystrophy. J Clin Med. Feb 6, 2022;11(3):856. [doi: 10.3390/jcm11030856] [Medline: 35160307]

18. Bertini S, Picariello M, Gorini M, et al. Telemonitoring in chronic ventilatory failure: a new model of survellaince, a pilot study. Monaldi Arch Chest Dis. Jun 2012;77(2):57-66. [doi: 10.4081/monaldi.2012.153] [Medline: 23193842]

19. Trucco F, Pedemonte M, Racca F, et al. Tele-monitoring in paediatric and young home-ventilated neuromuscular patients: a multicentre case-control trial. J Telemed Telecare. Aug 2019;25(7):414-424. [doi: 10.1177/1357633X18778479] [Medline: 29865934]

20. Onofri A, Pavone M, De Santis S, et al. Telemedicine in children with medical complexity on home ventilation during the COVID-19 pandemic. Pediatr Pulmonol. Jun 2021;56(6):1395-1400. [doi: 10.1002/ppul.25289] [Medline: 33524228]

21. Groh WJ, Bhakta D, Tomaselli GF et al. 2022 HRS expert consensus statement on evaluation and management of arrhythmic risk in neuromuscular disorders. Heart Rhythm. 2022 Oct;19(10):e61-e120. doi: 10.1016/j.hrthm.2022.04.022. Epub 2022 Apr 29. PMID: 35500790.

22. Kono T, Ogimoto A, Nishimura K, et al. Cardiac resynchronization therapy in a young patient with Duchenne muscular dystrophy. Int Med Case Rep J. 2015;8:173-175. [doi: 10.2147/IMCRJ.S87512]

23. Fayssoil A, Lazarus A, Wahbi K, et al. Cardiac implantable electronic devices in tracheotomized muscular dystrophy patients: safety and risks. Int J Cardiol. 2016;222:975-977. [doi: 10.1016/j.ijcard.2016.08.040] [Medline: 27526372]

24. Villa C, Auerbach SR, Bansal N, et al. Current practices in treating cardiomyopathy and heart failure in Duchenne Muscular Dystrophy (DMD): understanding care practices in order to optimize DMD heart failure through ACTION. Pediatr Cardiol. 2022;43(5):977-985. [doi: 10.1007/s00246-021-02807-7] [Medline: 35024902]

25. Ryan TD, Jefferies JL, Sawnani H, et al. Implantation of the HeartMate II and HeartWare left ventricular assist devices in patients with Duchenne muscular dystrophy: lessons learned from the first applications. ASAIO J. 2014;60(2):246-248. [doi: 10.1097/MAT.0000000000000050] [Medline: 24577373]

26. Iodice F, Testa G, Averardi M, et al. Implantation of a left ventricular assist device as a destination therapy in Duchenne muscular dystrophy patients with end stage cardiac failure: management and lessons learned. Neuromuscul Disord. 2015;25(1):19-23. [doi: 10.1016/j.nmd.2014.08.008] [Medline: 25444433]

27. Perri G, Filippelli S, Adorisio R, et al. Left ventricular assist device as destination therapy in cardiac end-stage dystrophinopathies: midterm results. J Thorac Cardiovasc Surg. 2017;153(3):669-674. [doi: 10.1016/j.jtcvs.2016.08.016] [Medline: 27692952]

28. Stoller D, Araj F, Amin A, et al. Implantation of a left ventricular assist device to provide long-term support for end-stage Duchenne muscular dystrophy-associated cardiomyopathy. ESC Heart Fail. 2017;4(3):379-383. [doi: 10.1002/ehf2.12160]

29. Jeannet PY, Aminian K, Bloetzer C, Najafi B, Paraschiv-Ionescu A. Continuous monitoring and quantification of multiple parameters of daily physical activity in ambulatory Duchenne muscular dystrophy patients. Eur J Paediatr Neurol. Jan 2011;15(1):40-47. [doi: 10.1016/j.ejpn.2010.07.002] [Medline: 20719551]

30. Kimura S, Ozasa S, Nomura K, Yoshioka K, Endo F. Estimation of muscle strength from actigraph data in Duchenne muscular dystrophy. Pediatr Int. Oct 2014;56(5):748-752. [doi: 10.1111/ped.12348] [Medline: 24689787]

31. Davidson ZE, Ryan MM, Kornberg AJ, Walker KZ, Truby H. Strong correlation between the 6-minute walk test and accelerometry functional outcomes in boys with Duchenne muscular dystrophy. J Child Neurol. Mar 2015;30(3):357-363. [doi: 10.1177/0883073814530502] [Medline: 24762862]

32. Le Moing AG, Seferian AM, Moraux A, et al. A movement monitor based on magneto-inertial sensors for non-ambulant patients with Duchenne muscular dystrophy: a pilot study in controlled environment. PLoS One. 2016;11(6):e0156696. [doi: 10.1371/journal.pone.0156696] [Medline: 27271157]

33. Fowler EG, Staudt LA, Heberer KR, et al. Longitudinal community walking activity in Duchenne muscular dystrophy. Muscle Nerve. Mar 2018;57(3):401-406. [doi: 10.1002/mus.25743] [Medline: 28692198]

34. van der Geest A, Essers JM, Bergsma A, Jansen M, de Groot IJ. Monitoring daily physical activity of upper extremity in young and adolescent boys with Duchenne muscular dystrophy: a pilot study. Muscle Nerve. Mar 2020;61(3):293-300. [doi: 10.1002/mus.26763] [Medline: 31742708]

35. Killian M, Buchowski MS, Donnelly T, et al. Beyond ambulation: measuring physical activity in youth with Duchenne muscular dystrophy. Neuromuscul Disord. Apr 2020;30(4):277-282. [doi: 10.1016/j.nmd.2020.02.007] [Medline: 32291149]

36. Arteaga D, Donnelly T, Crum K, et al. Assessing physical activity using accelerometers in youth with Duchenne muscular dystrophy. J Neuromuscul Dis. 2020;7(3):331-342. [doi: 10.3233/JND-200478] [Medline: 32417792]

37. Kaslow JA, Sokolow AG, Donnelly T, et al. Spirometry correlates with physical activity in patients with Duchenne muscular dystrophy. Pediatr Pulmonol. Apr 2023;58(4):1034-1041. [doi: 10.1002/ppul.26289] [Medline: 36571207]

38. Vilozni D, Bar-Yishay E, Gur I et al. Computerized respiratory muscle training in children with Duchenne muscular dystrophy. Neuromuscul Disord. May 1994;4(3):249-255. [doi: 10.1016/0960-8966(94)90026-4] [Medline: 7919973]

39. Hashimoto Y, Ushiba J, Kimura A et al. Change in brain activity through virtual reality-based brain-machine communication in a chronic tetraplegic subject with muscular dystrophy. BMC Neurosci. Sep 16, 2010;11(1):117. [doi: 10.1186/1471-2202-11-117] [Medline: 20846418]

40. Malheiros SR, da Silva TD, Favero FM, et al. Computer task performance by subjects with Duchenne muscular dystrophy. Neuropsychiatr Dis Treat. 2016;12:41-48. [doi: 10.2147/NDT.S87735] [Medline: 26766911]

41. Capelini CM, Silva TD, Tonks J et al. Improvements in motor tasks through the use of smartphone technology for individuals with Duchenne muscular dystrophy. Neuropsychiatr Dis Treat. 2017;13:2209-2217. [doi: 10.2147/NDT.S125466] [Medline: 28860778]

42. Heutinck L, Jansen M, van den Elzen Y et al. Virtual reality computer gaming with dynamic arm support in boys with Duchenne muscular dystrophy. J Neuromuscul Dis. 2018;5(3):359-372. [doi: 10.3233/JND-180307] [Medline: 29991140]

43. Massetti T, Favero FM, Menezes L et al. Achievement of virtual and real objects using a short-term motor learning protocol in people with Duchenne muscular dystrophy: a crossover randomized controlled trial. Games Health J. 2018;7(2):107-115. [doi: 10.1089/g4h.2016.0088] [Medline: 29608336]

44. de Freitas BL, da Silva TD, Crocetta TB, et al. Analysis of different device interactions in a virtual reality task in individuals with Duchenne muscular dystrophy—a randomized controlled trial. Front Neurol. 2019;10:24. [doi: 10.3389/fneur.2019.00024] [Medline: 30761066]

45. Quadrado VH, Silva TD, Favero FM et al. Motor learning transfer from virtual to real environments in Duchenne muscular dystrophy. Disabil Rehabil Assist Technol. 2019;14(1):12-20. [Medline: 29124971]

46. Correa AG, Klein AN, Lopes RD. Augmented reality musical system for rehabilitation of patients with Duchenne muscular dystrophy. In: Kheng TY, editor. Rehabilitation Engineering. IntechOpen; 2009:13-36. [doi: 10.5772/7395] ISBN: 9789535164159.

47. Fayssoil A, Orlikowski D, Nardi O, Annane D. Pacemaker implantation for sinus node dysfunction in a young patient with Duchenne muscular dystrophy. Congest Heart Fail. 2010;16(3):127-128. [doi: 10.1111/j.1751-7133.2009.00129.x] [Medline: 20557333] (Deleted).
